# Supplementary material for: Development of graphitic carbon nitride quantum dots-based oxygen self-sufficient platforms for enhanced corneal crosslinking
Source: Nat Commun. 2024 Jun 29;15:5508. doi: 10.1038/s41467-024-49645-8 (PMC11217369; doi:10.1038/s41467-024-49645-8)
Supplement: Supplementary file 1 — Supplementary Information [file 41467_2024_49645_MOESM1_ESM.docx]

**Supplementary Information**

**Development of graphitic carbon nitride quantum dots-based oxygen self-sufficient platforms for enhanced corneal crosslinking**

Mei Yang^1,2#,*^, Tingting Chen^2#^, Xin Chen^1#^, Hongxian Pan^1#^, Guoli Zhao^1#^, Zhongxing Chen^1^, Nan Zhao^3^, Qianfang Ye^2^, Ming Chen^1^, Shenrong Zhang^2^, Rongrong Gao^2^, Keith M. Meek^4^, Sally Hayes^4^, Xiaowei Ma^5^, Xin Li^2^, Yue Wu^1^, Yiming Zhang^6^, Na Kong^6^, Wei Tao^6^, Xingtao Zhou^1*^, Jinhai Huang^1*^

^1^ Eye Institute and Department of Ophthalmology, Eye & ENT Hospital, Fudan University; NHC Key laboratory of Myopia and Related Eye Diseases; Key Laboratory of Myopia and Related Eye Diseases, Chinese Academy of Medical Sciences, Shanghai Research Center of Ophthalmology and Optometry, Shanghai, China.

^2^ School of Ophthalmology and Optometry and Eye Hospital, Wenzhou Medical University, Wenzhou, Zhejiang 325027, China

^3^ School of Chemical Engineering, Northeast Electric Power University, Jilin 132000, China

^4^ School of Optometry and Vision Sciences, Cardiff University; Cardiff Institute for Tissue Engineering and Repair School of Pharmacy and Pharmaceutical Sciences, Cardiff University, Redwood Building, King Edward VII Avenue, Cardiff CF10 3NB, UK

^5^ School of Environmental Science and Engineering, Nanjing University of Information Science & Technology, Nanjing, 210044, China

^6^ Center for Nanomedicine and Department of Anesthesiology, Brigham and Women’s Hospital, Harvard Medical School, Boston, MA 02115, USA

^*^ Corresponding authors.

E-mail: meiyang@fudan.edu.cn (M.Y.), xingtaozhou@fudan.edu.cn (X.Z.); jinhaihuang@fudan.edu.cn (J.H.).

^#^ These authors contributed equally.

**
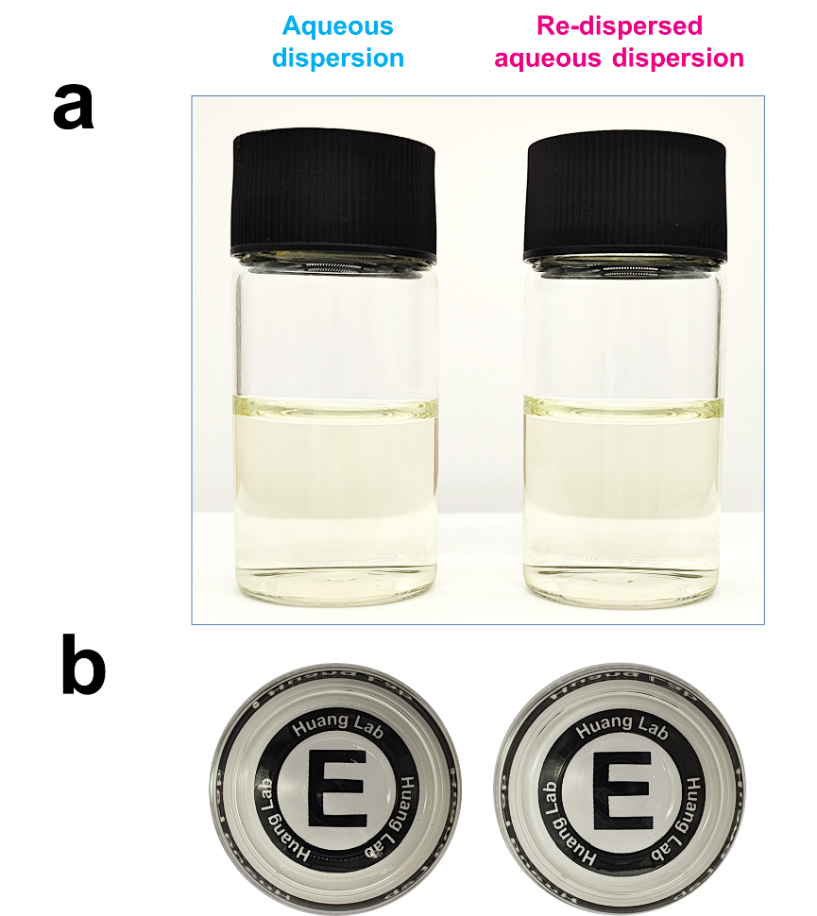
**

**Supplementary Figure 1.** Photographs of the g-C_3_N_4_ QDs aqueous dispersion and re-dispersed aqueous dispersion.

**
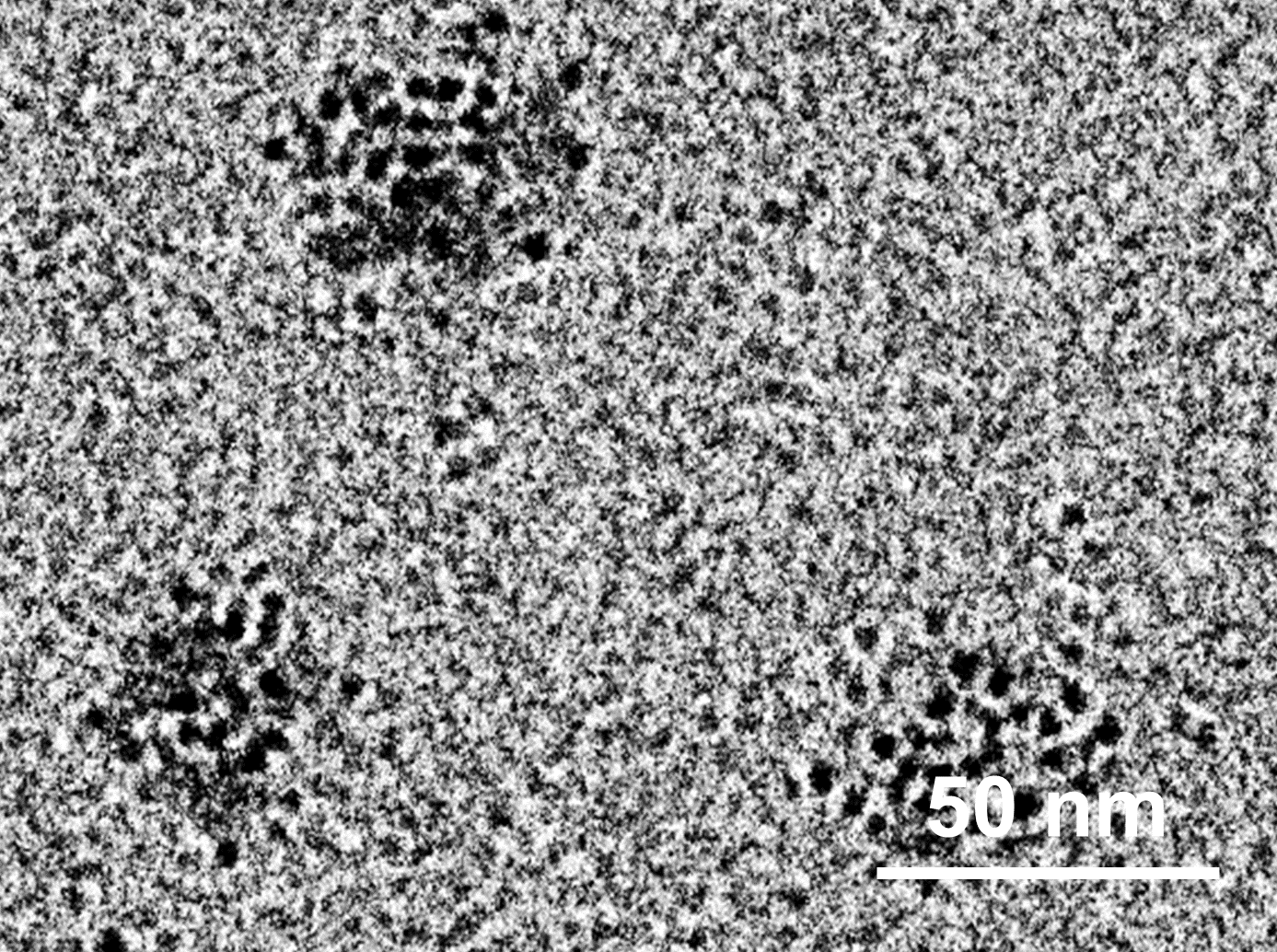
**

**Supplementary Figure 2.** TEM image of g-C_3_N_4_ QDs.

**
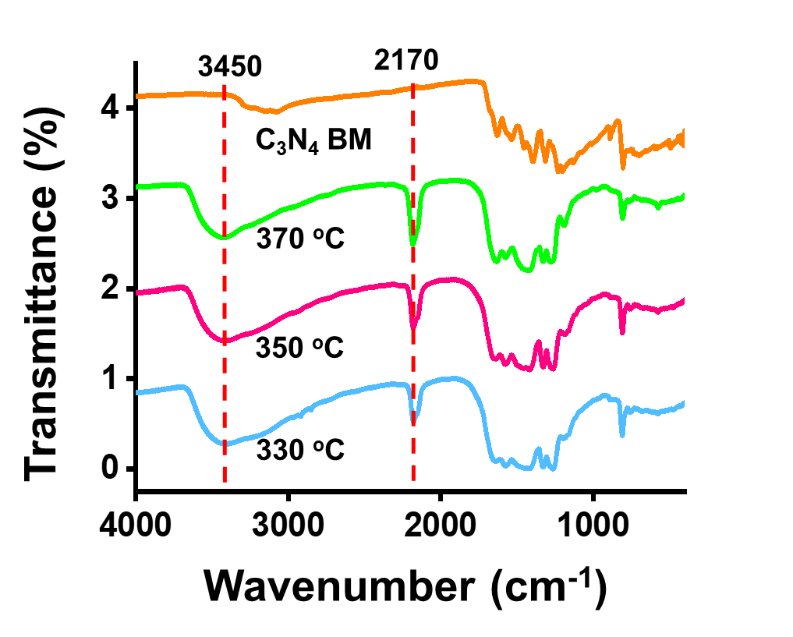
**

**Supplementary Figure 3.** FTIR spectra of g-C_3_N_4_ QDs obtained at different temperature as well as g-C_3_N_4_ BM.


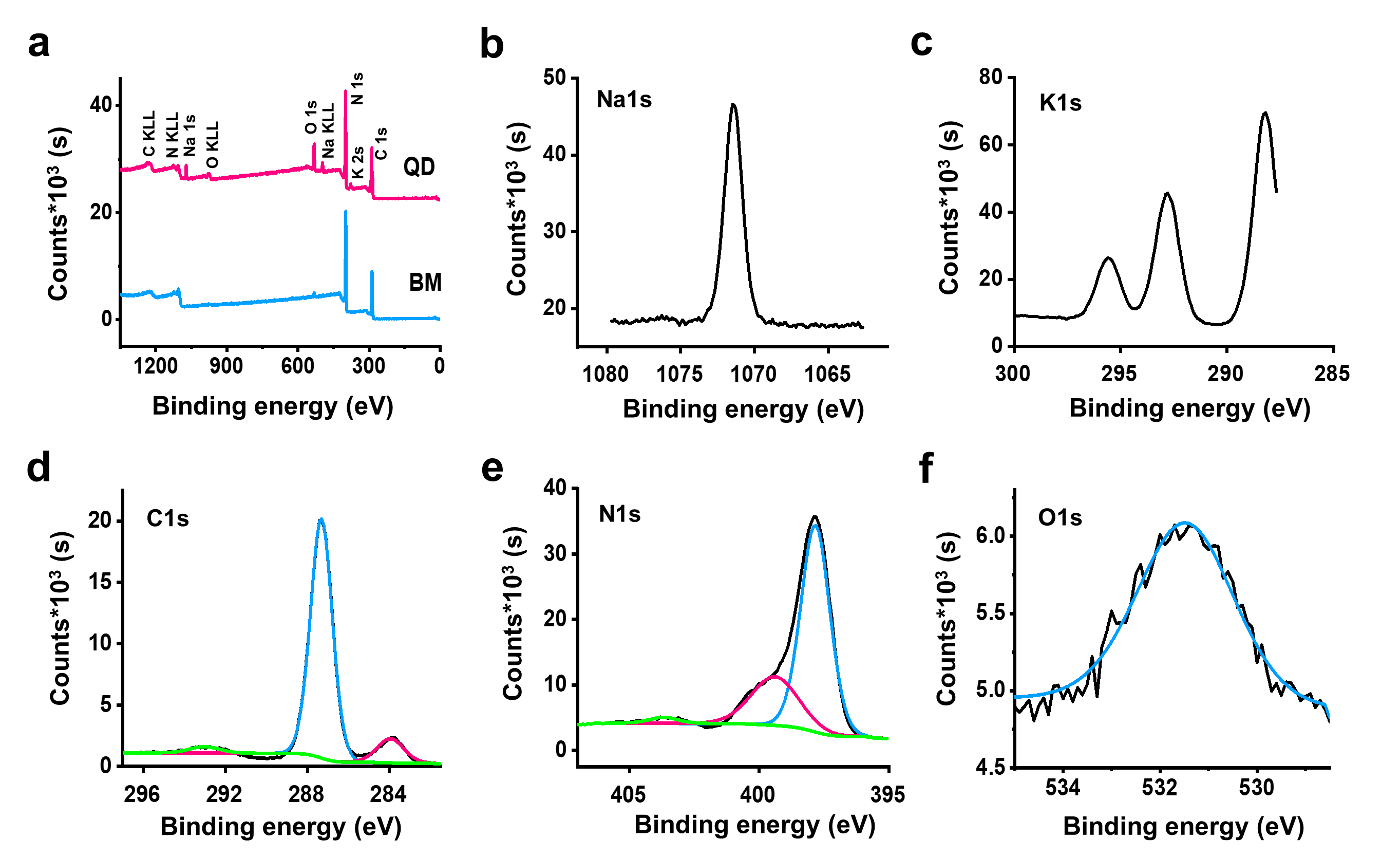


**Supplementary Figure 4.** XPS spectra of the g-C_3_N_4_ QDs obtained at 350 ^o^C and g-C_3_N_4_ BM. (a) Scan spectra of g-C_3_N_4_ QDs and g-C_3_N_4_ BM. (b) Na1s spectra of g-C_3_N_4_ QDs. (c) K1s spectra of g-C_3_N_4_ QDs. (d) C1s spectra of g-C_3_N_4_ BM. (e) N1s spectra of g-C_3_N_4_ BM. (f) O1s spectra of g-C_3_N_4_ BM.


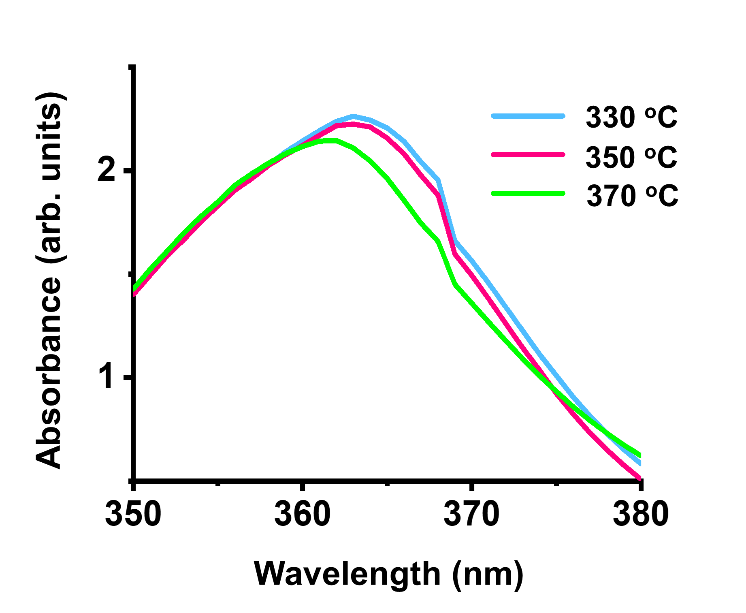


**Supplementary Figure 5.** UV–visible absorbance spectra of the g-C_3_N_4_ QDs obtained at different temperatures.


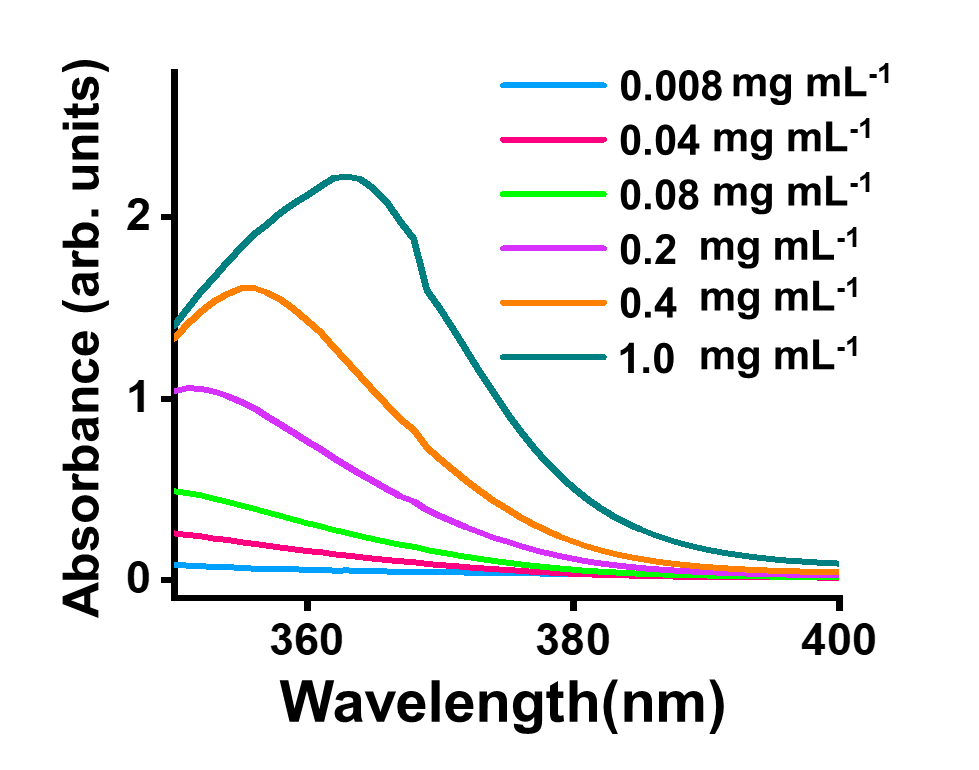


**Supplementary Figure 6.** UV–visible absorbance spectra of the g-C_3_N_4_ QDs obtained at 350 ^o^C with different concentrations.

**
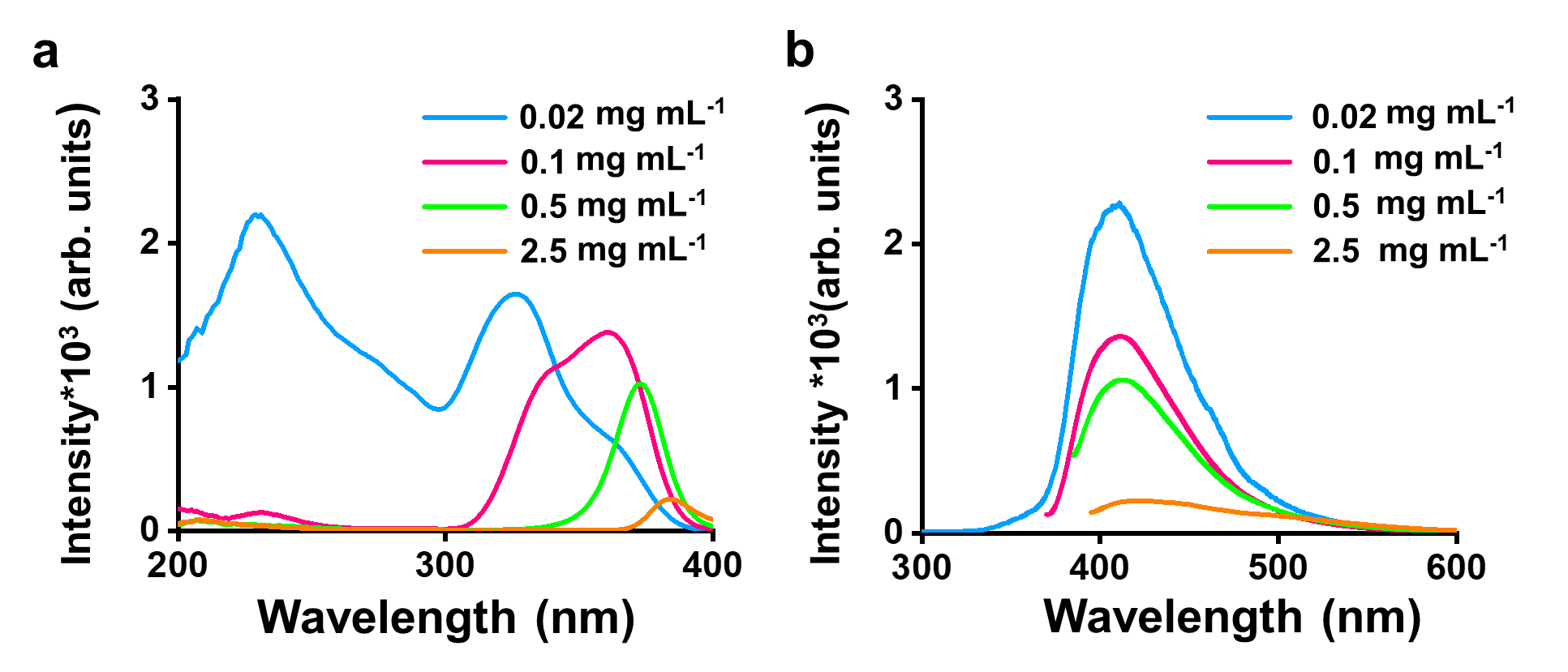
**

**Supplementary Figure 7.** Excitation and emission spectra of g-C_3_N_4_ QDs obtained at 350 ^o^C with different concentrations.


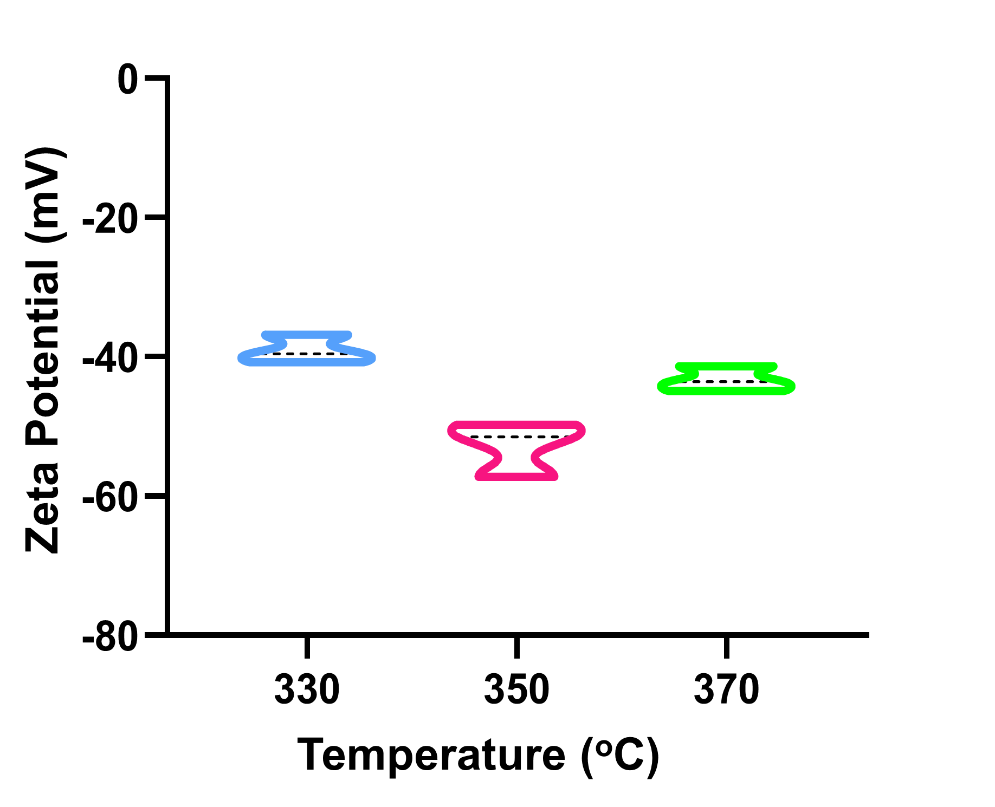


**Supplementary Figure 8.** Zeta potential of g-C_3_N_4_ QDs synthesized under different temperatures (mean ± SD, n=3).


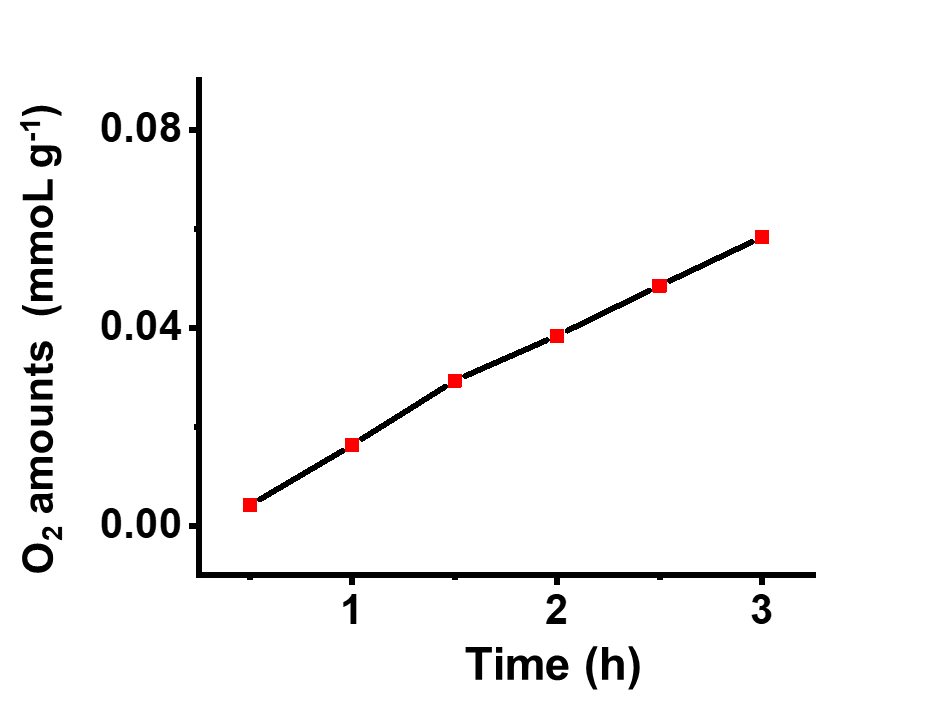


**Supplementary Figure 9.** Photocatalytic O_2_ evolution profile of g-C_3_N_4_ QDs obtained at 350 ^o^C under 365 nm UVA irradiation with an intensity of 180 mW cm^-2^ for a duration of 3 h.


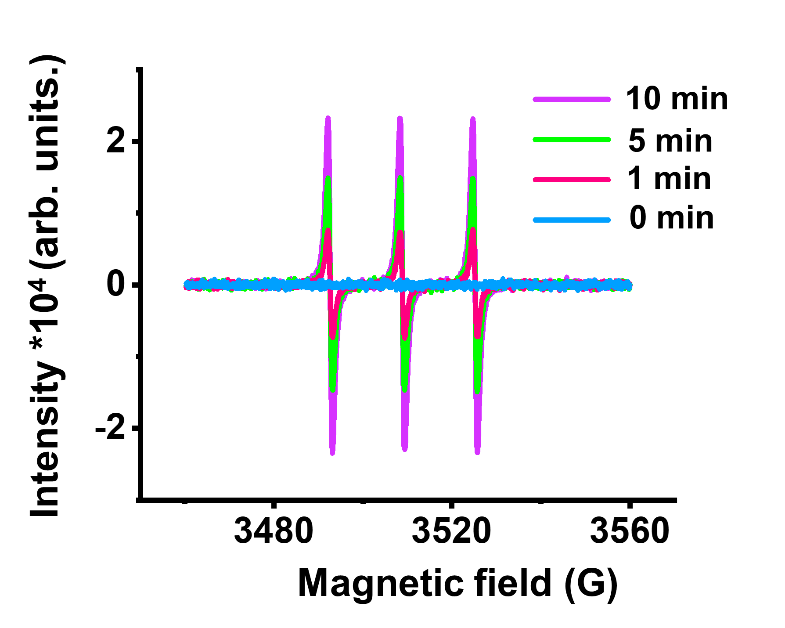


**Supplementary Figure 10.** Electron spin resonance (ESR) spectra of singlet oxygen generated by g-C_3_N_4_ QDs under 365 nm UVA irradiation with an intensity of 180 mW cm^-2^, captured at various time intervals (0, 1, 5, and 10 min).


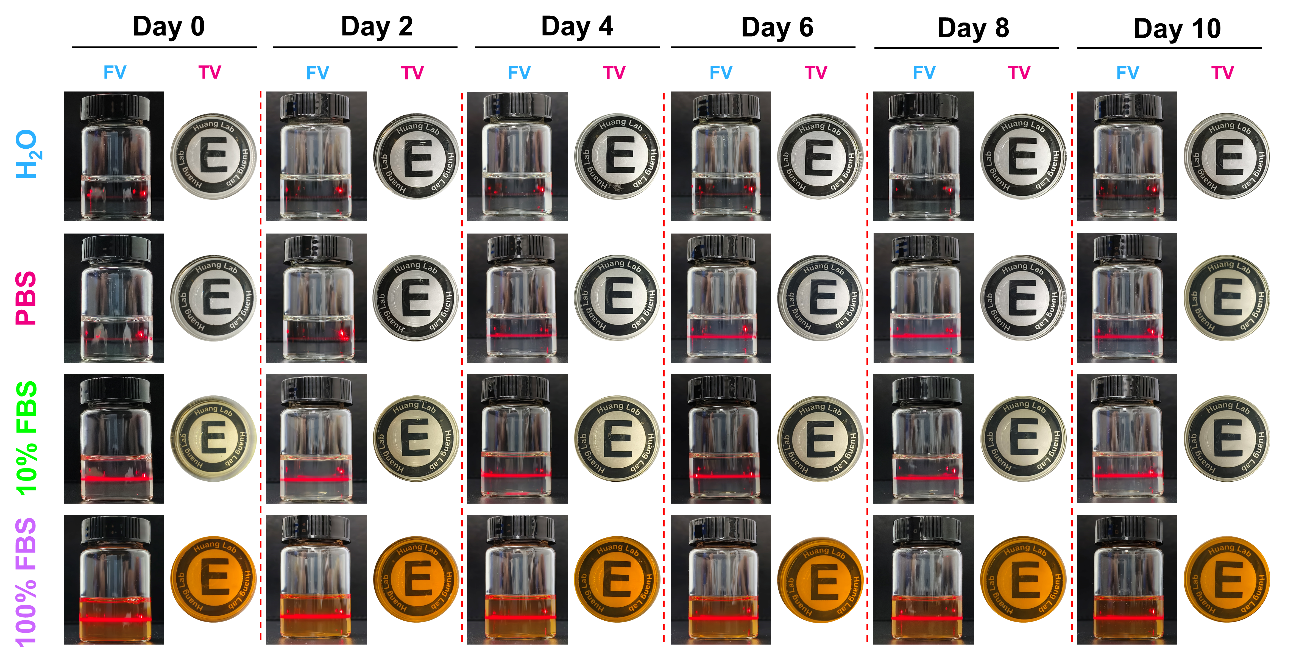


**Supplementary Figure 11.** The stability evaluation of the synthesized g-C_3_N_4_ QDs dispersed in deionized water (H_2_O), PBS, 10% fetal bovine serum (FBS) aqueous solution (10% FBS), and 100% FBS (FV: Front view, TV: Top view).


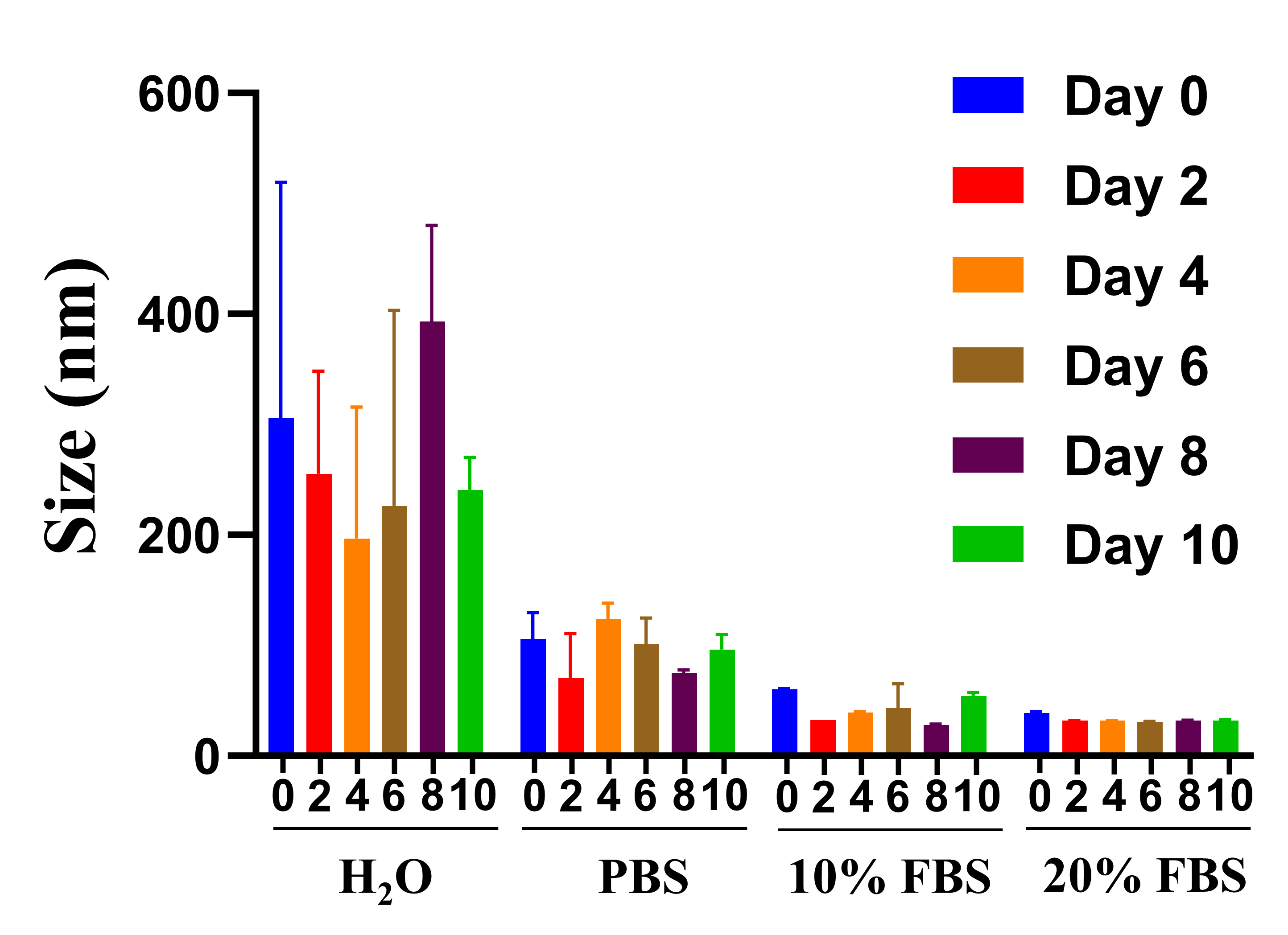


**Supplementary Figure 12.** The particle size distribution statistics patterns of the g-C_3_N_4_ QDs dispersed in deionized water (H_2_O), PBS, 10% fetal bovine serum (FBS) aqueous solution (10% FBS), and 100% FBS solution over a period of 10 days (mean ± SD, n=3, two-way ANOVA multiple comparison test, no marked P-value in the figure indicates no statistically significant difference between groups (p>0.05)).


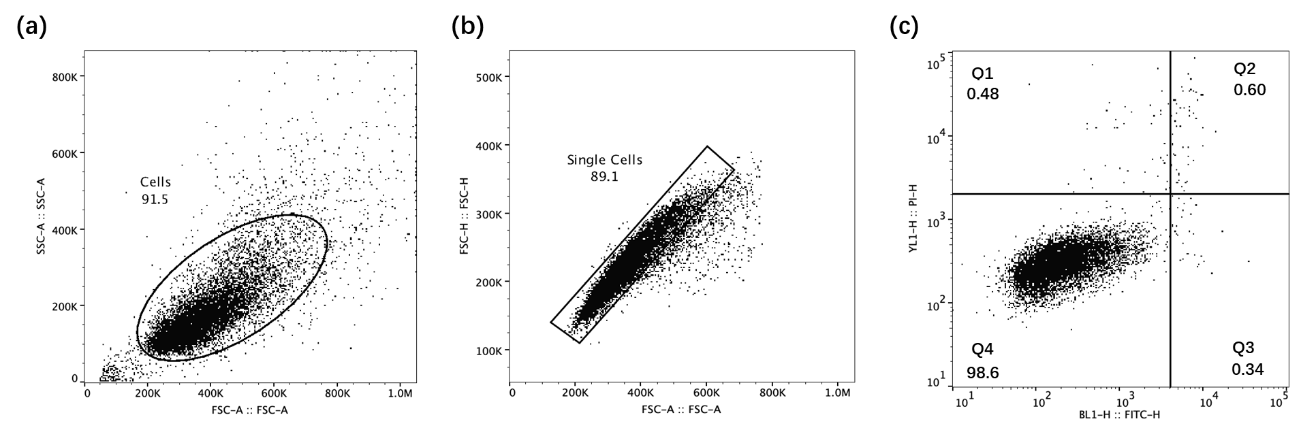


**Supplementary Figure 13.** A figure exemplifying the gating strategy for the flow cytometry process. a, b. Selective cell gating. c. Analysis the data (Q1: (AnnexinV-FITC)-/PI+, necrosis cells (post-apoptotic necrosis or late apoptosis), Q2: AnnexinV+FITC)+/PI+, late apoptptic cells, Q3: (AnnexinV-FITC)+/PI-, early apoptptic cells, Q4: (AnnexinV-FITC)-/PI-, living cells).

**
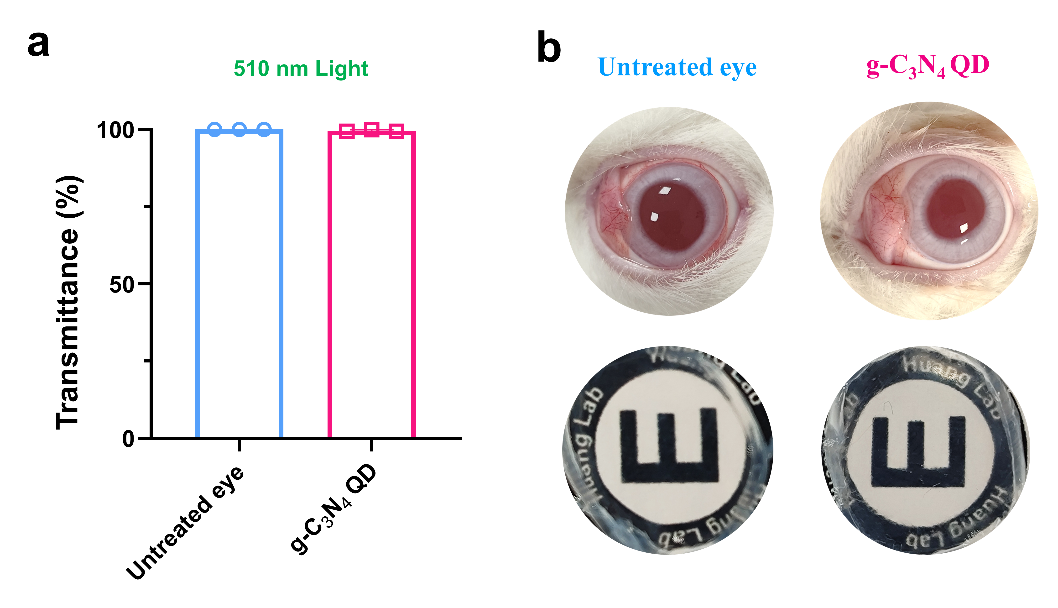
**

**Supplementary Figure 14.** The comparison of the transparence of the deepithelialized cornea presoaked for 30 min using 2.5 mg mL^-1^ g-C_3_N_4_ QDs aqueous dispersion with untreated cornea as control group (Con). (a) transmittance of the cornea with light wavelength controlled at 510 nm (mean ± SD, n=3, two-way ANOVA multiple comparison test, no marked P-value in the figure indicates no statistically significant difference between groups (p>0.05)). (b)Photographs of the corneas with different conditions.


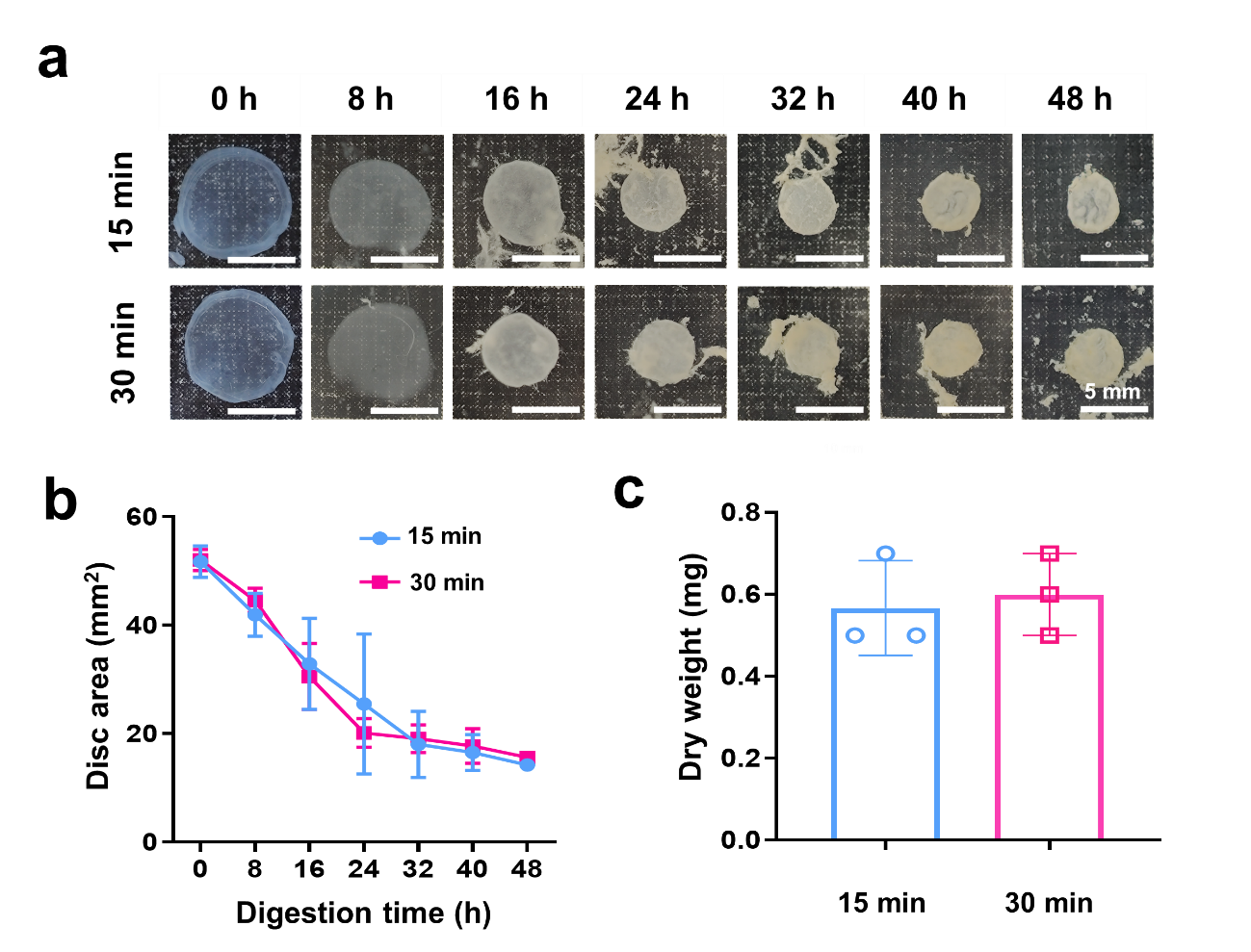


**Supplementary Figure 15.** The in vivo A-CXL evaluation with UVA intensity controlled at 6 mW cm^-2^ and different irradiation time. (a) The photos depicting the digestion behavior of the cornea in the presence of collagenase II. (b)A statistic analysis on the change in disc area over time (mean ± SD, n=3, two-way ANOVA multiple comparison test, the absence of a marked P-value in the figure signifies that there is no statistically significant difference between the groups (p>0.05)). (c) The dry weight of the residual cornea at 48 h (mean ± SD, n=3, two-way ANOVA multiple comparison test, the lack of a marked P-value in the figure denotes no statistically significant difference between the groups (p>0.05)).

**
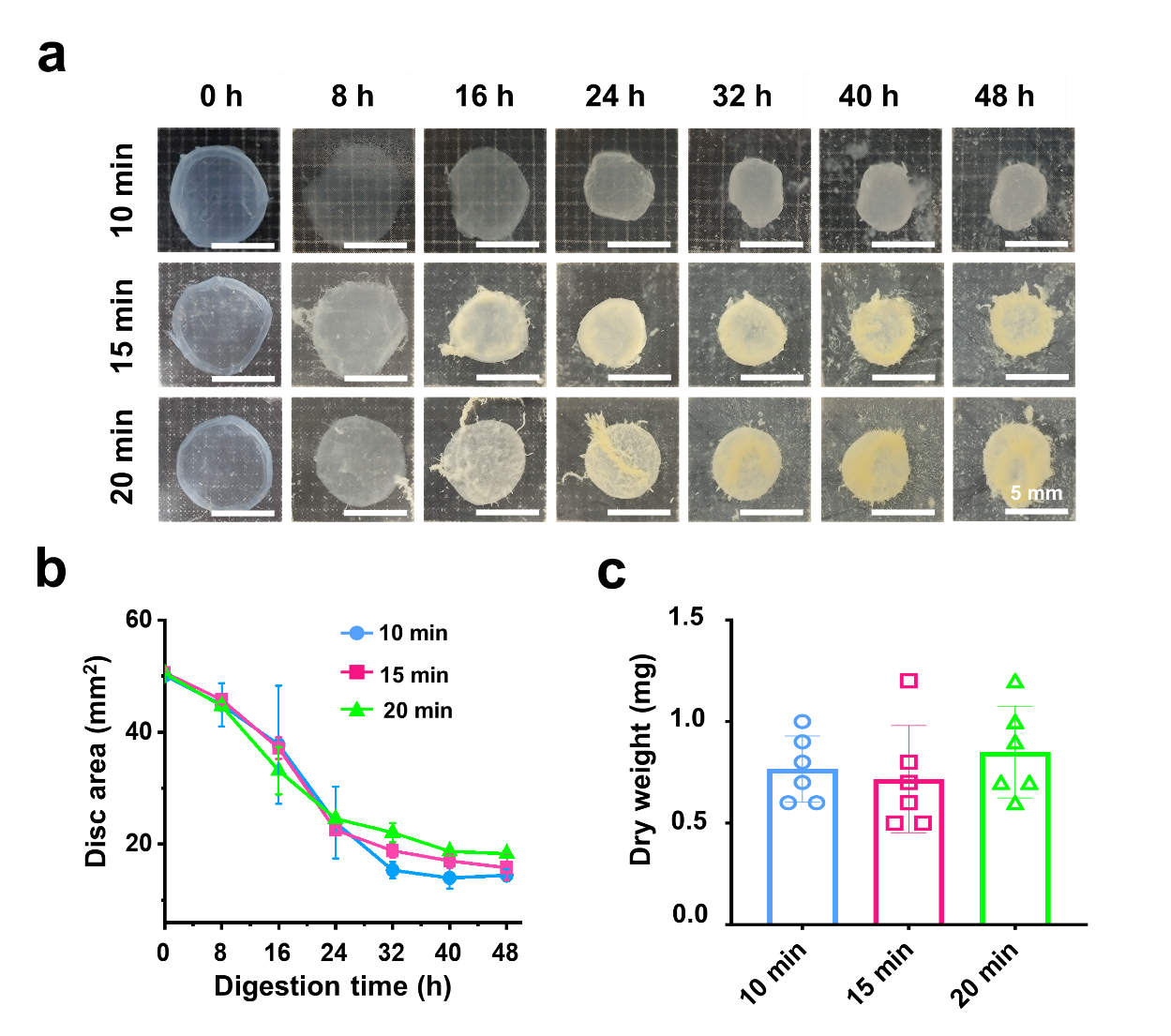
**

**Supplementary Figure 16.** The in vivo A-CXL evaluation with UVA intensity controlled at 9 mW cm^-2^ and different irradiation time. (a) The photos depicting the digestion behavior of the cornea in the presence of collagenase II. (b) Quantifications of the disc area in disc area over time (mean ± SD, n = 6, two-way ANOVA multiple comparison test, no marked P-value in the figure indicates no statistically significant difference between groups (p>0.05)). (c) The dry weight of the residual cornea at 48 h (mean ± SD, n =6, two-way ANOVA multiple comparison test, no marked P-value in the figure indicates no statistically significant difference between groups (p>0.05)).


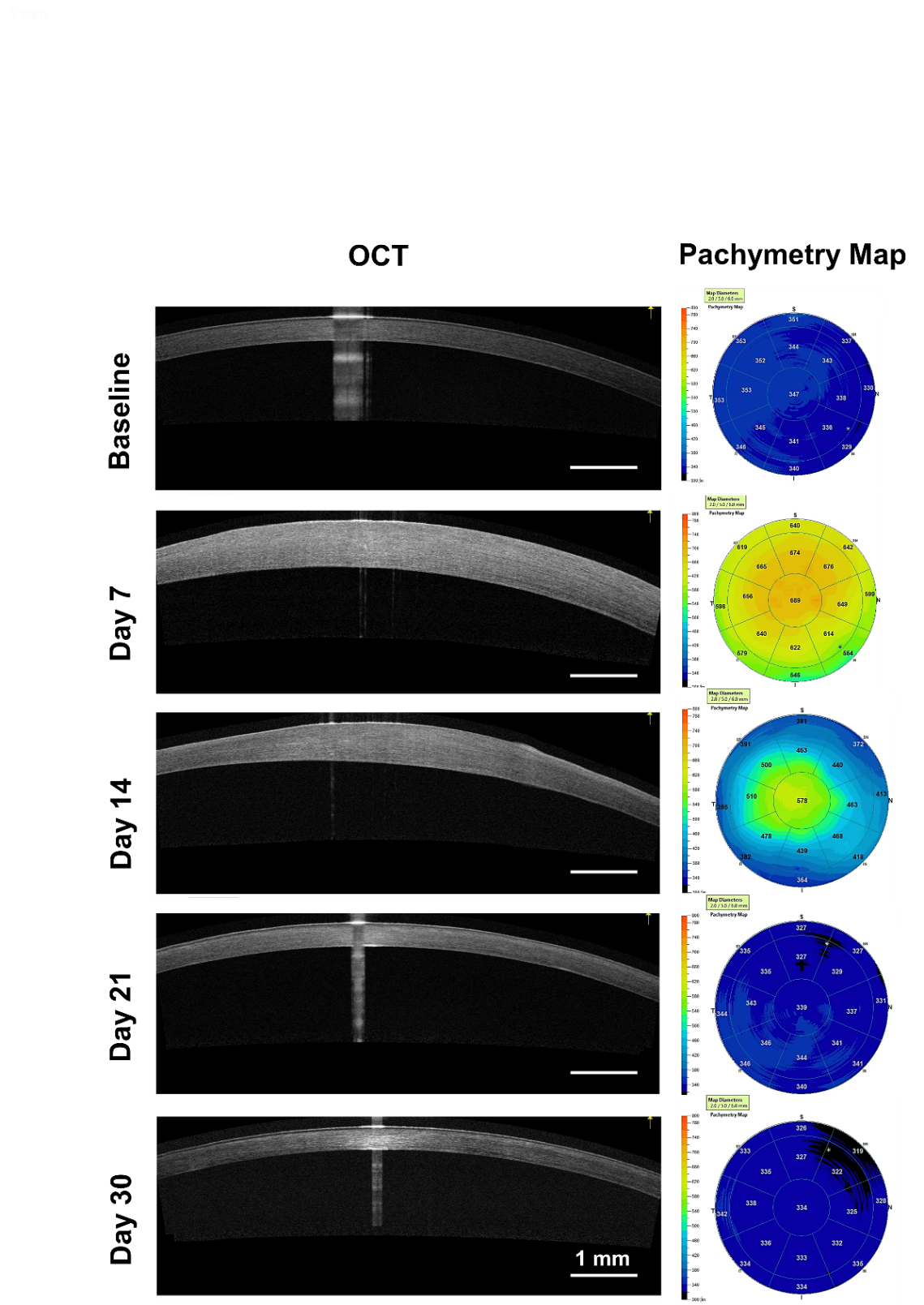


**Supplementary Figure 17.** OCT images of cornea in g-C_3_N_4_ QDs group at different time intervals after A-CXL (n=4, similar results).

**
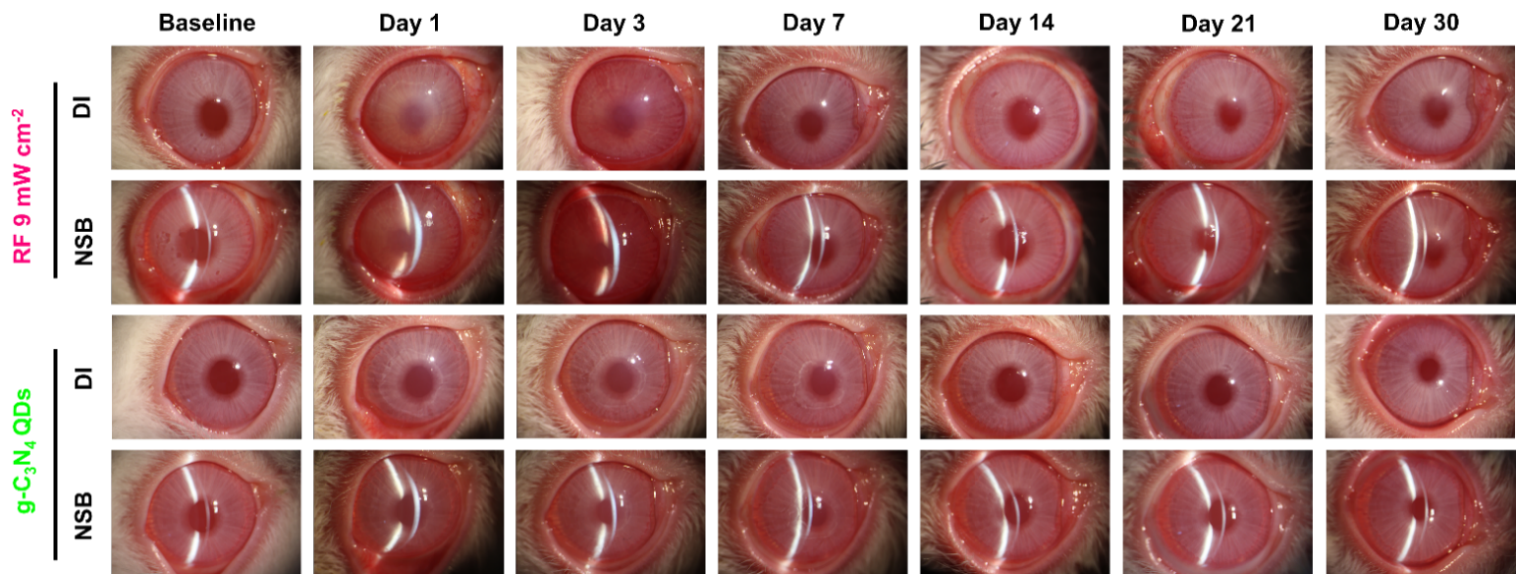
**

**Supplementary Figure 18.** The postoperative reaction and recovery condition of the rabbits’ eye after A-CXL with different photosensitizer (n=4, similar results). DI: The photographs were taken using diffuse illumination for observation of the anterior segment overview. NSB: The photographs were taken using narrow slit beam with background illumination for observation of corneal thickness.

**
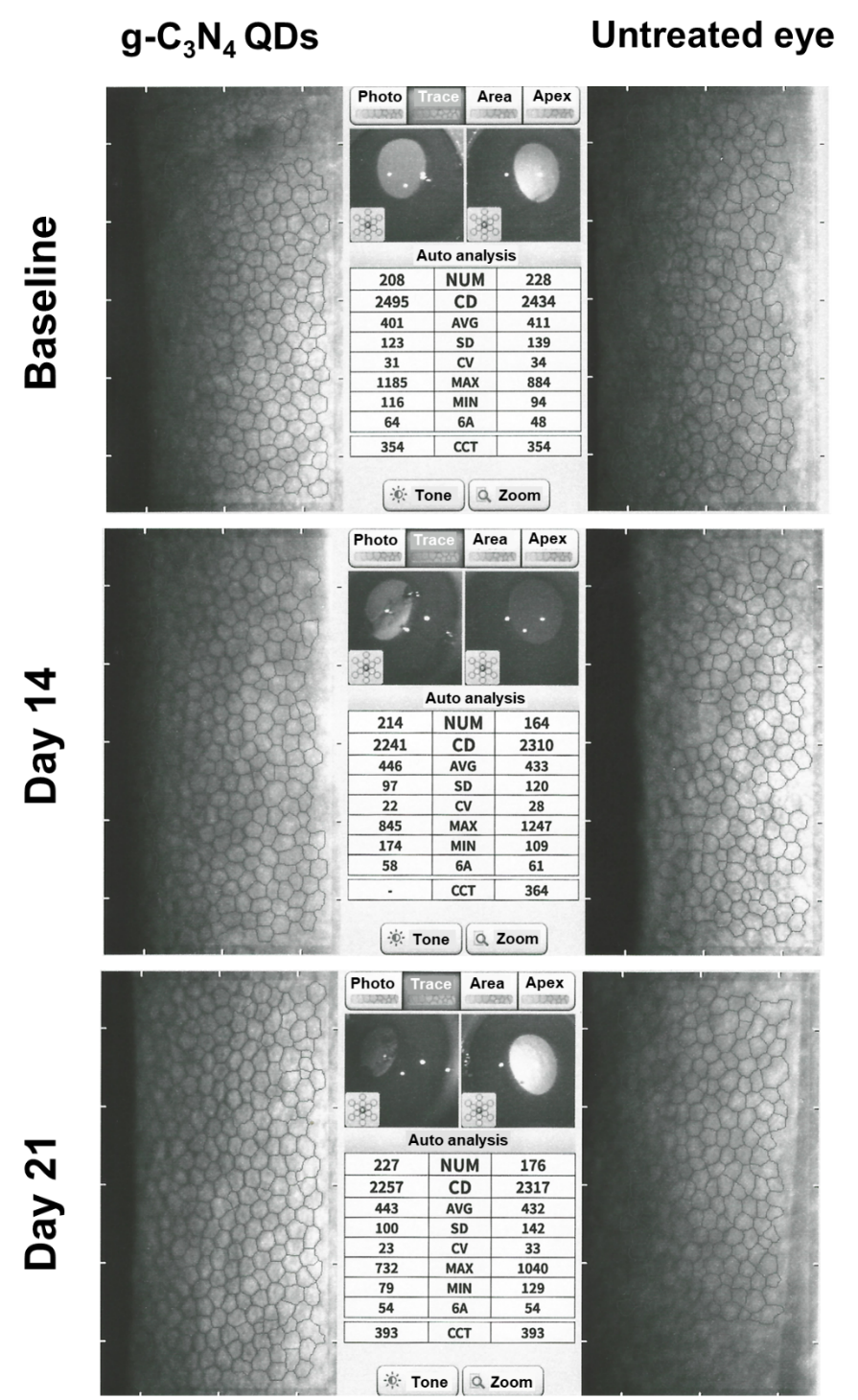
**

**Supplementary Figure 19.** The photos of the in vivo corneal endothelium in g-C_3_N_4_ QDs groups after A-CXL for different observation time (n=4, similar results).


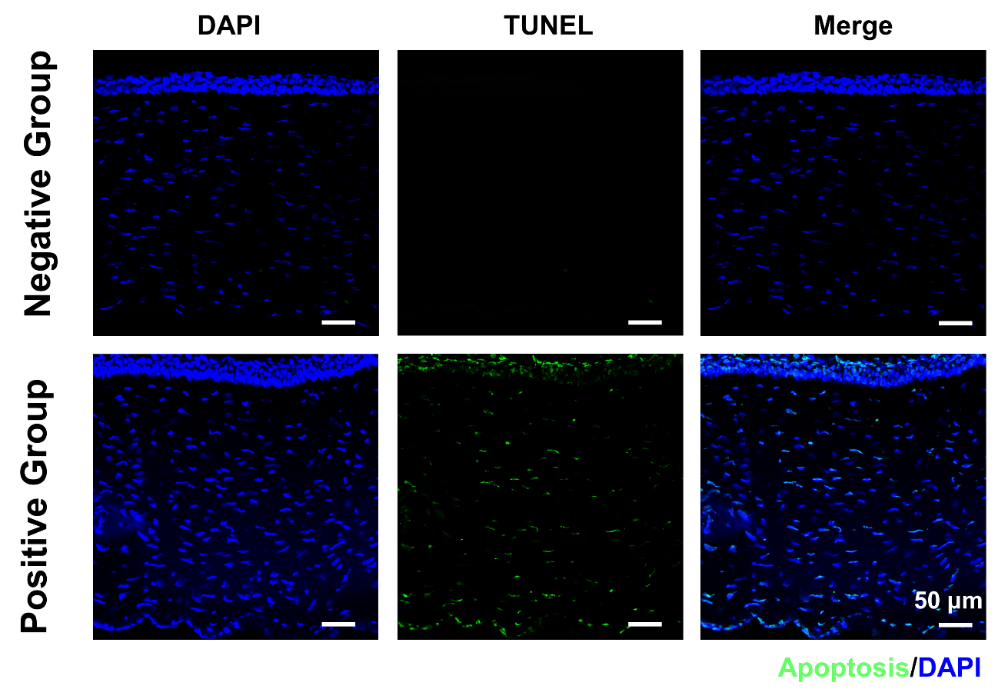


**Supplementary Figure 20.** The confocal images of negative group (normal tissue) and positive groups (DNase-treated) in TUNEL testing (n=3, similar results).


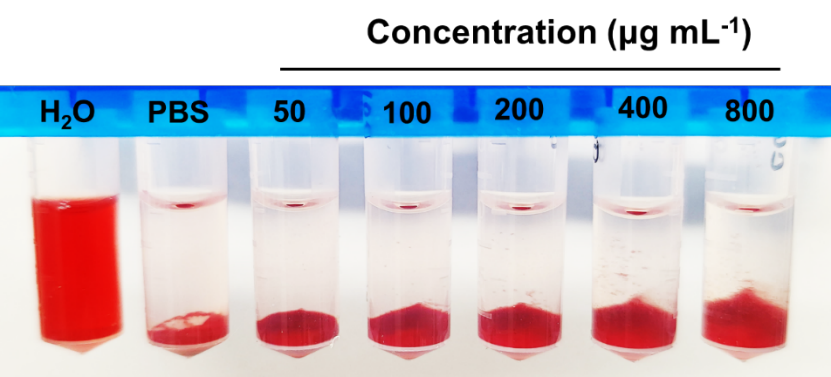


**Supplementary Figure 21.** The hemolytic reaction of red blood cells incubated with water, PBS, or g-C_3_N_4_ QDs for 2 h (n=5, similar results).


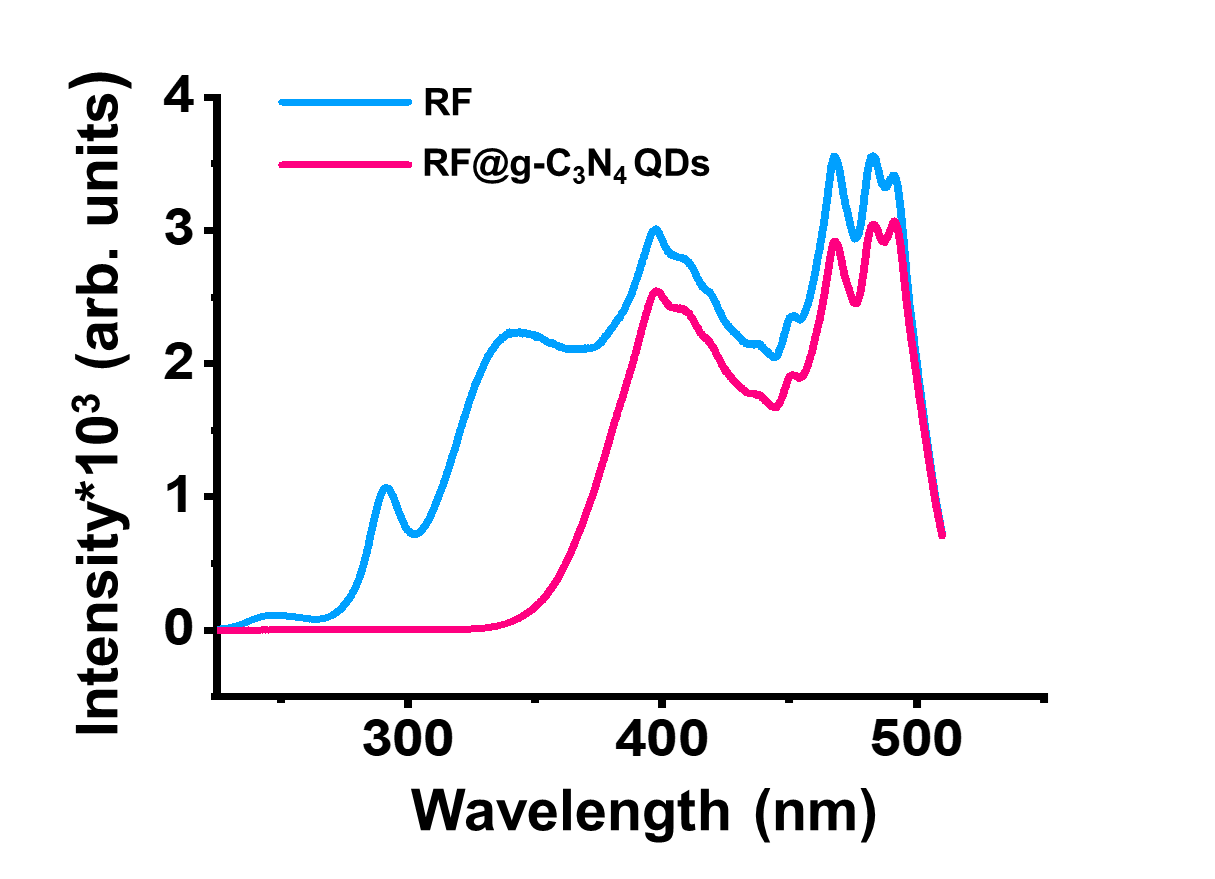


**Supplementary Figure 22.** The excitation spectra of RF and RF@g-C_3_N_4_ QDs.


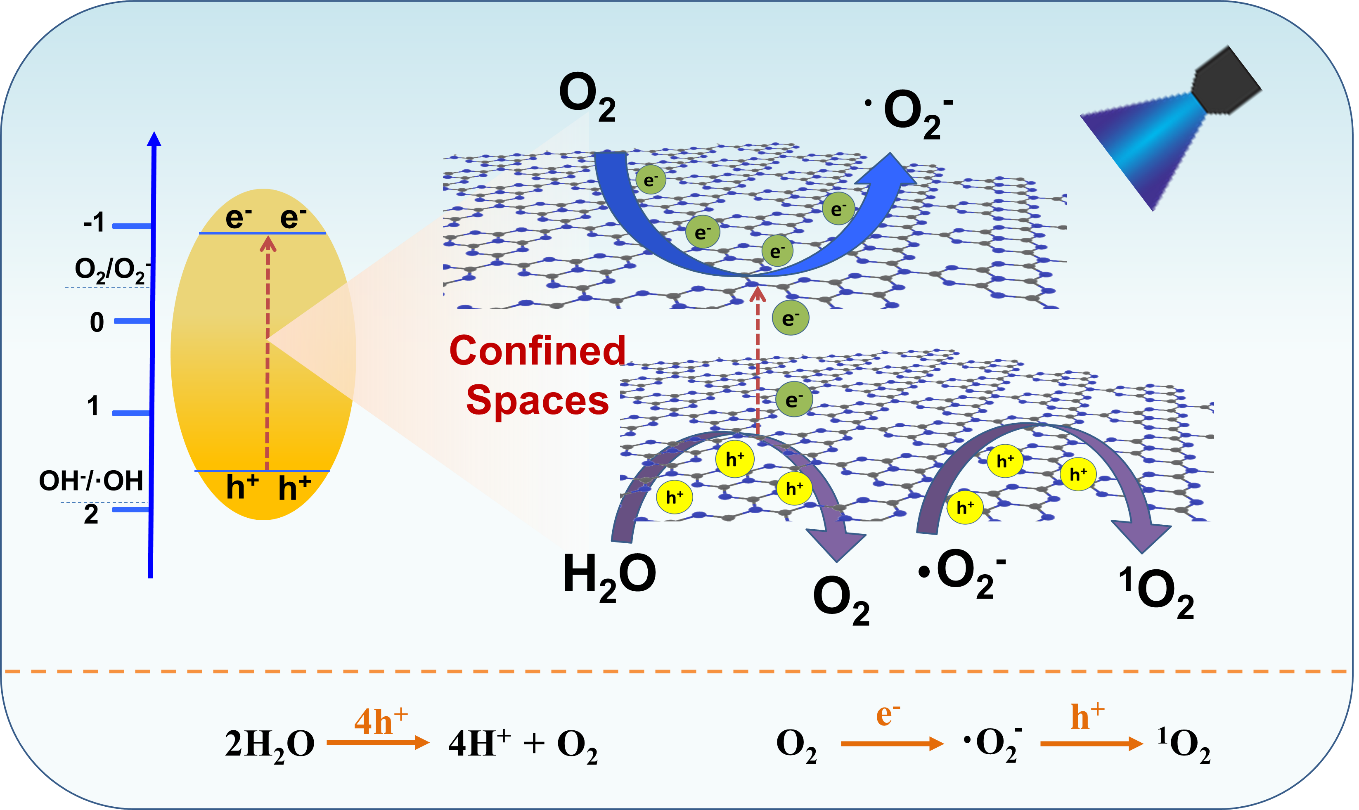


**Supplementary Figure 23.** The possible functional mechanism of g-C_3_N_4_ QDs in the process of A-CXL.
