# Supplementary material for: Development of graphitic carbon nitride quantum dots-based oxygen self-sufficient platforms for enhanced corneal crosslinking
Source: Nat Commun. 2024 Jun 29;15:5508. doi: 10.1038/s41467-024-49645-8 (PMC11217369; doi:10.1038/s41467-024-49645-8)
Supplement: Supplementary file 2 — Reporting Summary [file 41467_2024_49645_MOESM2_ESM.pdf]

nature portfolio

Corresponding author(s): Mei Yang, Xingtao Zhou, Jinhai Huang

Last updated by author(s): May 18, 2024

Reporting Summary

Nature Portfolio wishes to improve the reproducibility of the work that we publish. This form provides structure for consistency and transparency in reporting. For further information on Nature Portfolio policies, see our Editorial Policies and the Editorial Policy Checklist.

Statistics

For all statistical analyses, confirm that the following items are present in the figure legend, table legend, main text, or Methods section.

n/a	Confirmed	
☐	☒	The exact sample size (*n*) for each experimental group/condition, given as a discrete number and unit of measurement
☐	☒	A statement on whether measurements were taken from distinct samples or whether the same sample was measured repeatedly
☐	☒	The statistical test(s) used AND whether they are one- or two-sided<br>*Only common tests should be described solely by name; describe more complex techniques in the Methods section.*
☐	☒	A description of all covariates tested
☒	☐	A description of any assumptions or corrections, such as tests of normality and adjustment for multiple comparisons
☐	☒	A full description of the statistical parameters including central tendency (e.g. means) or other basic estimates (e.g. regression coefficient) AND variation (e.g. standard deviation) or associated estimates of uncertainty (e.g. confidence intervals)
☐	☒	For null hypothesis testing, the test statistic (e.g. *F*, *t*, *r*) with confidence intervals, effect sizes, degrees of freedom and *P* value noted<br>*Give P values as exact values whenever suitable.*
☒	☐	For Bayesian analysis, information on the choice of priors and Markov chain Monte Carlo settings
☒	☐	For hierarchical and complex designs, identification of the appropriate level for tests and full reporting of outcomes
☒	☐	Estimates of effect sizes (e.g. Cohen's *d*, Pearson's *r*), indicating how they were calculated

Our web collection on statistics for biologists contains articles on many of the points above.

Software and code

Policy information about availability of computer code

Data collection	The data were collected by analytical instrument of a Smartlab 9 powder Diffractometer, high-resolution transmission electron microscope (HRTEM, JEM2100F), Thermal Fisher Nicolet 6700 spectrometer, HITACHI U-3900 UV–visible spectrophotometry, Hitachi F-7000 Fluorescence Spectrophotometer, UVA light (ZF-20D Crypto UV analyzer), Dissolved oxygen analyzer (DOS-1703), Malvern Zetasizer equipment, Thermo Scientific Escalab 250 Xi spectrometer, BD Accuri™ C6 Flow Cytometer, Leica DM750, Leica DMI8 inverted fluorescence microscope, Instron 68TM-30, Optovue RTVue OCT, Instron 68TM-30, Corvis® ST, Slit lamp biomicroscopy, Specular microscope TOMEY,EM-4000, Agilent Synergy H1 microplate reader, Rayto Chemray420, Mindray BC-2800vet, OLYMPUS APX100 Standard.
Data analysis	Data analysis were performed by using BD Accuri™ C6 Plus, GraphPad Prism 8, Jade 6.5 , Casa2273 xps, Origin2021, imageJ v1.54f, Zen.

For manuscripts utilizing custom algorithms or software that are central to the research but not yet described in published literature, software must be made available to editors and reviewers. We strongly encourage code deposition in a community repository (e.g. GitHub). See the Nature Portfolio guidelines for submitting code & software for further information.

Data

Policy information about availability of data

All manuscripts must include a data availability statement. This statement should provide the following information, where applicable:

- Accession codes, unique identifiers, or web links for publicly available datasets
- A description of any restrictions on data availability
- For clinical datasets or third party data, please ensure that the statement adheres to our policy

All data supporting the findings described in this manuscript are available within the paper and the supplementary information. The source data are provided with this paper, or available from the corresponding author upon request.

Research involving human participants, their data, or biological material

Policy information about studies with human participants or human data. See also policy information about sex, gender (identity/presentation), and sexual orientation and race, ethnicity and racism.

Reporting on sex and gender	N/A
Reporting on race, ethnicity, or other socially relevant groupings	N/A
Population characteristics	N/A
Recruitment	N/A
Ethics oversight	N/A

Note that full information on the approval of the study protocol must also be provided in the manuscript.

Field-specific reporting

Please select the one below that is the best fit for your research. If you are not sure, read the appropriate sections before making your selection.

☒ Life sciences ☐ Behavioural & social sciences ☐ Ecological, evolutionary & environmental sciences

For a reference copy of the document with all sections, see nature.com/documents/nr-reporting-summary-flat.pdf

Life sciences study design

All studies must disclose on these points even when the disclosure is negative.

Sample size	No statistical methods was used to predetermine the samples size. The sample sizes were determined based on previous related studies and be sufficient to obtain statistically significant difference between experimental groups(n=3-7). For in vivo studies, each group contains more then 3 for evaluating the statistical significance. These sample sizes also represent the standard practice for publication in this field and were described in figure legends.
Data exclusions	No data were excluded from the analysis.
Replication	The sample size in cell and animal experimental groups were ≥ 3 to ensure replicability. From the measured results, the data showed high similarity within the same testing group, and the replicability was good in each group.
Randomization	Rabbits/cells were randomly allocated to different experimental groups before treatment. They were treated and maintained in the same environment and randomly allocated to each group.
Blinding	The investigators were blinded to group allocation during experiments, data collection and analysis

Reporting for specific materials, systems and methods

We require information from authors about some types of materials, experimental systems and methods used in many studies. Here, indicate whether each material, system or method listed is relevant to your study. If you are not sure if a list item applies to your research, read the appropriate section before selecting a response.

Materials & experimental systems

n/a	Involved in the study	
☒	☐	Antibodies
☐	☒	Eukaryotic cell lines
☒	☐	Palaeontology and archaeology
☐	☒	Animals and other organisms
☒	☐	Clinical data
☒	☐	Dual use research of concern
☒	☐	Plants

Methods

n/a	Involved in the study	
☒	☐	ChIP-seq
☐	☒	Flow cytometry
☒	☐	MRI-based neuroimaging

Eukaryotic cell lines

Policy information about cell lines and Sex and Gender in Research

Cell line source(s)	Human cornea epithelial cells (HCECs) were purchased from the BeNa Culture Collection (BNCC, BNCC337836). Human retinal microvascular endothelial cells (hRMECs) were purchased from the BeNa Culture Collection (BNCC, BNCC363743). Adult Retinal Pigment Epithelial cell line-19 (ARPE-19) was purchased from the BeNa Culture Collection (BNCC, BNCC337713)
Authentication	These cell lines were authenticated by the supplier using STR analysis.
Mycoplasma contamination	All cell lines were tested negative for mycoplasma contamination by the supplier BNCC.
Commonly misidentified lines (See ICLAC register)	No commonly misidentified cell lines were used in the study.

Animals and other research organisms

Policy information about studies involving animals; ARRIVE guidelines recommended for reporting animal research, and Sex and Gender in Research

Laboratory animals	Male New Zealand white rabbits (weight, 2.0–2.5 kg, 4 months) were purchased from Danyang Changyi experimental animal breeding Co., Ltd., and raised with free access to food and water throughout the test period.
Wild animals	This study did not involve wild animals.
Reporting on sex	Sex was not considered in the study.
Field-collected samples	This study did not involve samples collected from the field.
Ethics oversight	The animal study was approved by the Ethical Committee of EYE and ENT Hospital of Fudan University(IACUCDWZX-2023-026), and in accordance with the ARVO Statement for the Use of Animals in Ophthalmic and Vision Research.

Note that full information on the approval of the study protocol must also be provided in the manuscript.

Plants

Seed stocks	N/A
Novel plant genotypes	N/A
Authentication	N/A

Flow Cytometry

Plots

Confirm that:

☒ The axis labels state the marker and fluorochrome used (e.g. CD4-FITC).

☒ The axis scales are clearly visible. Include numbers along axes only for bottom left plot of group (a 'group' is an analysis of identical markers).

☒ All plots are contour plots with outliers or pseudocolor plots.

☒ A numerical value for number of cells or percentage (with statistics) is provided.

Methodology

Sample preparation	HCEC cells were first seeded on 96-well plates and incubated for 24 h at 37 °C. The medium was then exchanged for fresh medium containing g-C3N4 QDs with different concentrations. After 24 h of incubation, cells were collected by trypsin digestion, washed with PBS and resuspended in 300 µL 1X binding buffer. Then, 4 µL Annexin V-FITC and 1.5 µL PI were added in sequence. After being kept in dark for another 15 min, all samples were analyzed using an Accuri C6 flow cytometer to evaluate cell viability.
Instrument	BD Accuri™ C6 Flow Cytometer
Software	BD Accuri™ C6 Plus
Cell population abundance	No Cell Sorting was performed in this study.
Gating strategy	Determine the boundary between negative and positive fluorescence parameters based on FITC and PI fluorescence values, and determine the cross gate. In typical experiments, cells can be divided into four subgroups: living cells are double negative (Annexin V-FITC -/PI -); early apoptotic cells are single positive for Annexin V-FITC (Annexin V-FITC+/PI -); late apoptotic cells are double positive for Annexin V-FITC and PI (Annexin V-FITC+/PI+), while necrotic cells are single positive for PI (Annexin V-FITC -/PI+).

☐ Tick this box to confirm that a figure exemplifying the gating strategy is provided in the Supplementary Information.
